# Ecoregional Analysis of Nearshore Sea-Surface Temperature in the North Pacific

**DOI:** 10.1371/journal.pone.0030105

**Published:** 2012-01-11

**Authors:** Meredith C. Payne, Cheryl A. Brown, Deborah A. Reusser, Henry Lee

**Affiliations:** 1 Western Fisheries Research Center, United States Geological Survey, Newport, Oregon, United States of America; 2 Oregon State University, Corvallis, Oregon, United States of America; 3 Pacific Coastal Ecology Branch, Western Ecology Division, United States Environmental Protection Agency, Newport, Oregon, United States of America; University of Vigo, Spain

## Abstract

The quantification and description of sea surface temperature (SST) is critically important because it can influence the distribution, migration, and invasion of marine species; furthermore, SSTs are expected to be affected by climate change. To better understand present temperature regimes, we assembled a 29-year nearshore time series of mean monthly SSTs along the North Pacific coastline using remotely-sensed satellite data collected with the Advanced Very High Resolution Radiometer (AVHRR) instrument. We then used the dataset to describe nearshore (<20 km offshore) SST patterns of 16 North Pacific ecoregions delineated by the Marine Ecoregions of the World (MEOW) hierarchical schema. Annual mean temperature varied from 3.8°C along the Kamchatka ecoregion to 24.8°C in the Cortezian ecoregion. There are smaller annual ranges and less variability in SST in the Northeast Pacific relative to the Northwest Pacific. Within the 16 ecoregions, 31–94% of the variance in SST is explained by the annual cycle, with the annual cycle explaining the least variation in the Northern California ecoregion and the most variation in the Yellow Sea ecoregion. Clustering on mean monthly SSTs of each ecoregion showed a clear break between the ecoregions within the Warm and Cold Temperate provinces of the MEOW schema, though several of the ecoregions contained within the provinces did not show a significant difference in mean seasonal temperature patterns. Comparison of these temperature patterns shared some similarities and differences with previous biogeographic classifications and the Large Marine Ecosystems (LMEs). Finally, we provide a web link to the processed data for use by other researchers.

## Introduction

Considerable progress has been made in understanding ocean dynamics through the analysis of remotely-sensed sea surface temperature (hereafter SST) data [1], [2], [3], [4]. However, potential contamination by land signal and coastal weather often hampers efforts to compile a comprehensive nearshore SST dataset. This issue was encountered in the study by Blanchette et al. [5], which examined the relationship between temperature and species assemblages in rocky shores from southeast Alaska to Baja California, and in a study by Broitman et al. [6] of a smaller region along the coasts of Oregon and California. Both inquiries experienced an issue with missing pixels, forcing them to spatially average the SST data at their coastal sites. Despite these difficulties, Pearce et al. [7] found that monthly mean values of remotely-sensed nearshore SSTs were viable for examining seasonal patterns in the nearshore region.

These studies exemplify the use of nearshore SST records for a variety of marine-related research. A spatio-temporally continuous coastal SST dataset at a broad spatial scale can help address macroecological questions relating to biogeographical patterns, invasions, and climate change. At a biogeographic scale, temperature is a major factor affecting distributions of native and nonindigenous species (NIS) [8], [9], [10], [11]. One specific example of this type of application is predicting potential areas susceptible to invasion of NIS using environmental matching based on temperature [12], [13]. Furthermore, these types of climate studies are critical because nearshore environments and the distributions of organisms within them are expected to be highly influenced by climate change [6], [14], [15].

In nearshore regions, *in situ* SST measurements are typically made by moored buoys; however, while there is a paucity of SST data, the mooring sites and sampling intervals are irregular in both space and time. Hence, there is a need for a satellite-derived SST product capable of covering extensive areas of the coastline. Satellite remote-sensing observations have the advantage of extensive spatial coverage and high repeatability in relatively inaccessible regions that is not often possible with field observations. The trade-off, however, is that satellite data have lower spatial resolution compared with field measurements and therefore often cannot resolve more localized processes important in coastal areas. Furthermore, high-resolution image mosaics of an expansive area often consist of a massive amount of data, making them impractical for many users.

To address this data gap, a SST product capable of covering as much coastline as possible while keeping resolution and file size reasonable is needed. Therefore, we generated a consistent SST product of a known quality for the nearshore region of the North Pacific Basin based on SST measurements from the Advanced Very High Resolution Radiometer (AVHRR) Pathfinder data. The resulting tri-decadal, moderate-resolution dataset was used to evaluate nearshore SST, providing a regional-scale view of seasonal temperature patterns for the entire North Pacific coast. We provide sufficient background on the generation of the data and their quality for use by ecologists and biogeographers and to serve as a readily-available baseline for climate studies.

## Materials and Methods

### Study Area

**Figure 1:**
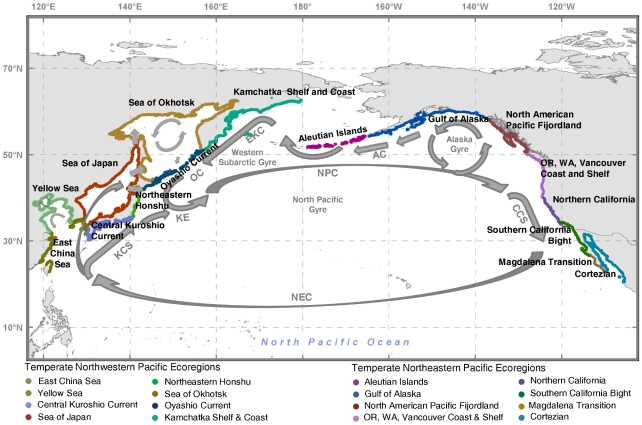
Temperate North Pacific realm, and the 16 MEOW ecoregions included in this paper. Major surface circulation pathways are labeled on the map and indicated with blue arrows, including the North Pacific Current (NPC), California Current System (CCS), North Equatorial Current (NEC), Kuroshio Current System (KCS), Kuroshio Extension (KE), Oyashio Current (OC), East Kamchatka Current (EKC).

In the effort to characterize nearshore ecosystems at a regional scale, we analyzed SST measurements by marine “ecoregions” as defined by The Nature Conservancy in their MEOW biogeographic schema [16]. The MEOW schema is a global hierarchical classification system of coastal zones that divides the world's coastal areas into 12 distinct marine “realms.” The realms are further broken down into 62 marine “provinces” which are further divided into 232 “ecoregions.” In generating this schema, the objective was to develop a “hierarchical system based on taxonomic configurations , influenced by evolutionary history, patterns of dispersal , and isolation”[17]. We focused the present analysis on the ecoregions within the Temperate North Pacific realm, which is comprised of four Provinces containing 17 individual ecoregions (Figure 1). Additionally, we used the breakout of the Northeast Pacific Region and Northwest Pacific Region as defined by Reusser and Lee [17] to distinguish between ecoregions on the two sides of the North Pacific. We limited our analysis to expansive coastal regions and therefore did not include the Puget Trough/Georgia Basin ecoregion in this analysis.

### SST Data Description

We selected the AVHRR-derived Pathfinder monthly-mean SST dataset [18] versions 5.0 (years 1985–2009) and 5.1 (years 1981–1985; PFSST V50 and V51, respectively) for its global coverage at moderate resolution (each grid cell measures approximately 4 km×4 km), long data record relative to other satellite missions, and substantial level of processing, including extensive calibration and atmospheric correction. We obtained the PFSST V50 “hierarchical data format scientific dataset” (HDF-SDS) data from the NASA JPL Physical Oceanography Distributed Active Archive Center (PO.DAAC) (ftp://podaac.jpl.nasa.gov/pub/sea_surface_temperature/avhrr/pathfinder/data_v5/) and the PFSST V51 data for 1981–1985 from NODC (ftp://data.nodc.noaa.gov/pub/data.nodc/pathfinder).

The PFSST V50 data were produced specifically for use in the analysis of global change and have improved resolution (from 9 km to 4 km) compared to PFSST V41 in coastal regions [19]. The PFSST data include a quality flag in which each SST grid cell is designated a value ranging from 0 (worst quality) to 7 (best quality). These quality flags convey the level of confidence attributed to the SST value calculated for each grid cell location. Level of confidence is evaluated on pixel-by-pixel performance based on a number of tests that estimate validity and consistency of brightness temperature readings, sun angle effects, and cloudiness, which are combined to establish an overall quality rating. While daytime data have a more pronounced diurnal warming effect, it has been shown that they are not necessarily inferior to nighttime data [20]. Therefore, we used the PFSST daytime series because daytime data were more abundant in the nearshore regions of interest, especially in areas that experience frequent evening ground fog, such as along the coasts of Washington and Oregon, USA.

### SST Data Processing Stream

Subsequent to downloading the Pathfinder data, the Marine Geospatial Ecology Tools v. 0.8 (MGET) extension was used to convert the PFSST data from its native HDF-SDS format to Environmental Systems Research Institute (ESRI) ArcGIS rasters [21]. The PFSST V41 dataset (the PFSST dataset preceding V50, which was retired in 2005) included a standard product called “best SST,” or “BSST,” which included only grid cells with quality flags greater than 3 [18]. For this study, we used the same quality threshold as BSST. The next step was to eliminate unlikely SST values (<−2.0°C) that occasionally appear in the subarctic or Arctic. The remaining data were scaled using the equation provided in the AVHRR metadata in order to obtain SST values in degrees Celsius.

As the focus of this research is on nearshore environments, we limited the study area to within 20 km of the coastline (Figure 1). Often, the grid cell closest to the coast was eliminated from the analysis due to a low quality rating related to land-contamination. We selected grid cells by counting four grid cells seaward of the coastline defined by the Global Self-consistent, Hierarchical, High-resolution Shoreline (GSHHS) dataset [22]. ArcGIS was used to isolate the grid cells in each monthly-mean SST raster. These steps were systematically repeated to generate a point shapefile of nearshore SST values—one for each month of each year for the interval of September 1981 to December 2009. The resulting dataset consisted of more than 2 million points representing individual spatial locations for each month stored in 340 ESRI shapefiles, which were aggregated into three smaller datasets based on month, MEOW provinces and ecoregions. These steps were automated using open source R statistical software [23] scripts. Synthesized data and scripts used in this analysis are available from Payne et al. [24] (http://pubs.usgs.gov/of/2010/1251/index.html).

### SST analysis using MEOW as a geographic framework

**Figure 2:**
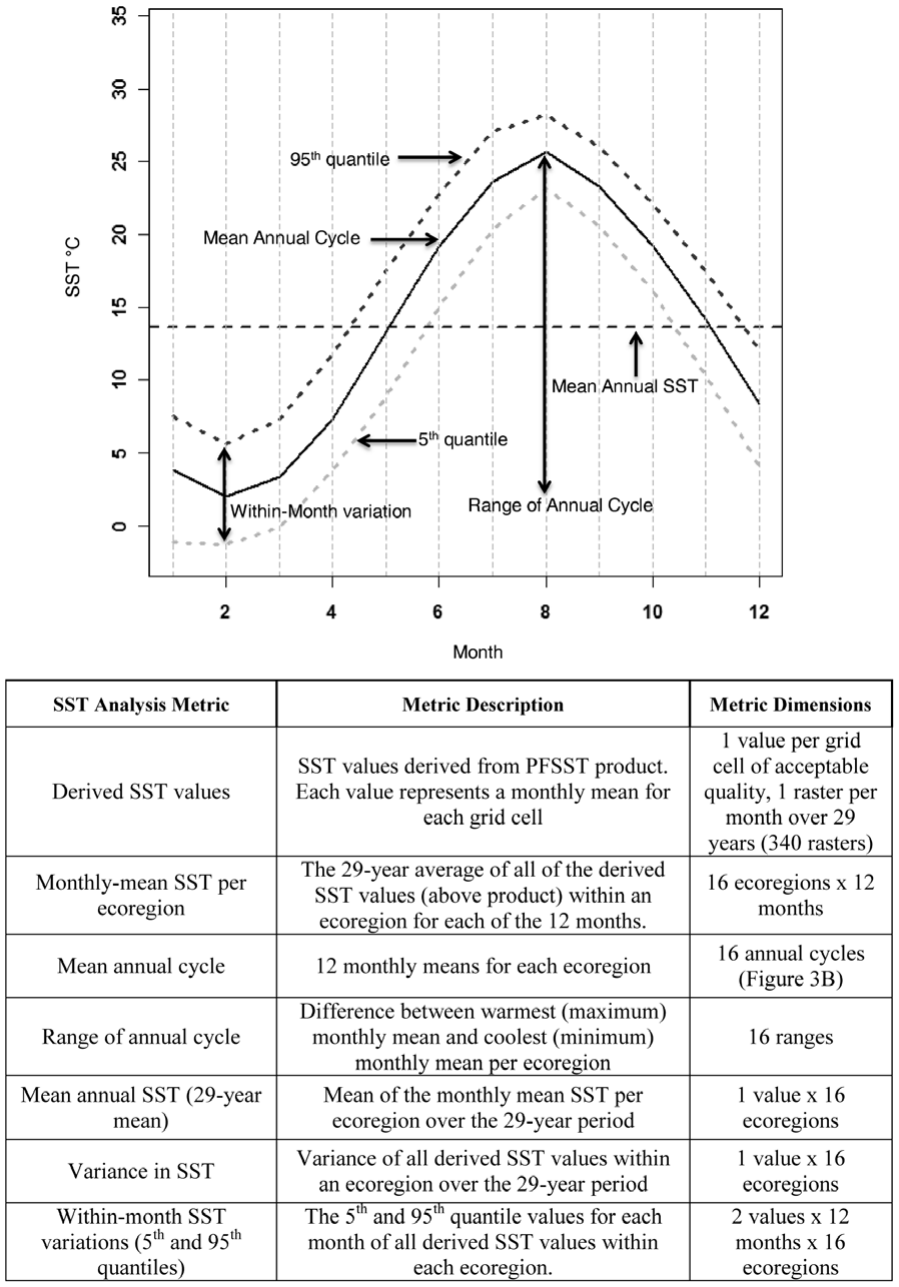
Annual cycle metrics analyzed in this study. Generalized illustration and tabular description of metrics used to evaluate the annual SST cycle in the North Pacific ecoregions. Individual, ecoregion-specific annual cycles are depicted in Figure 3.

**Figure 3:**
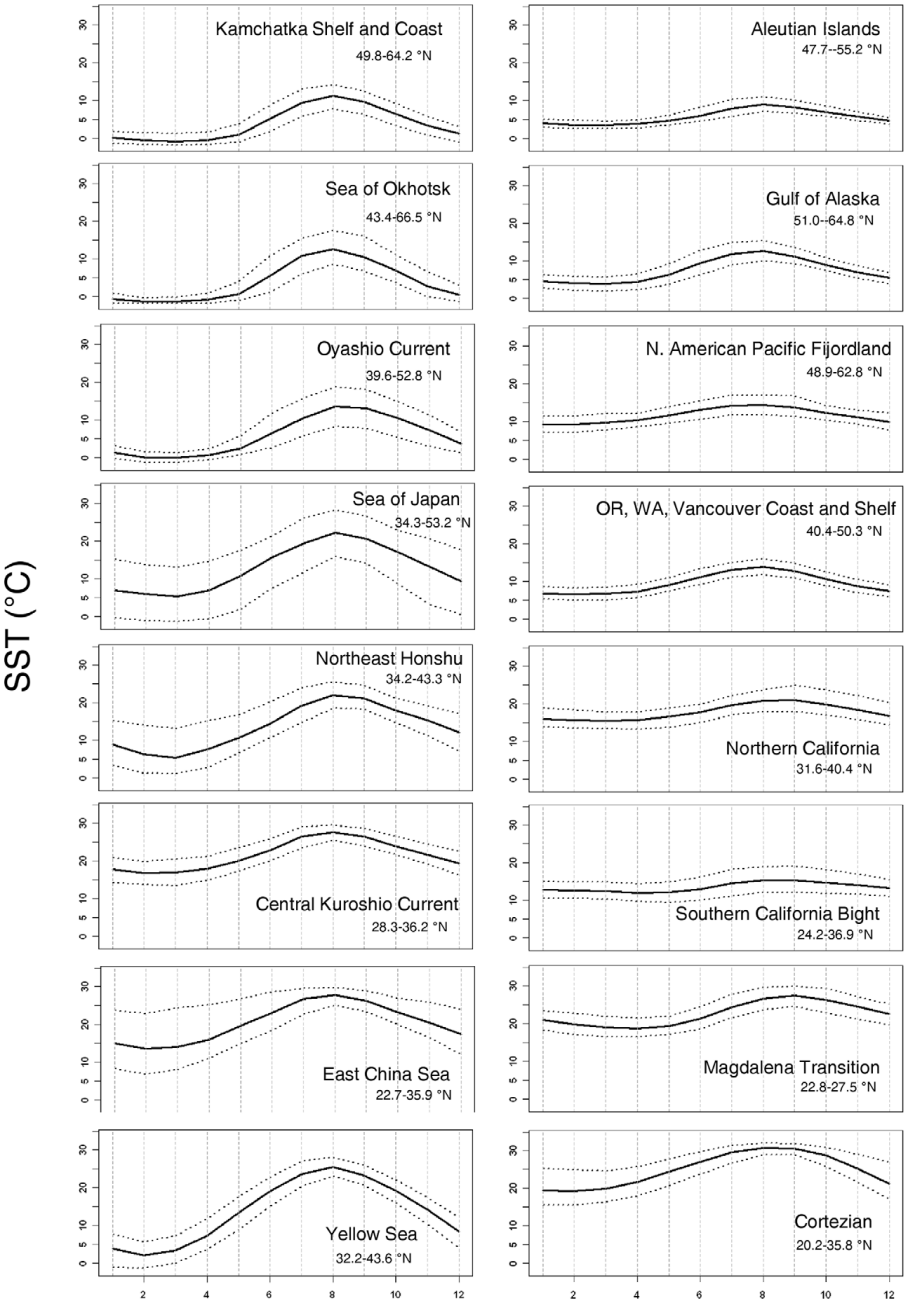
Monthly-mean SST in each of the Temperate North Pacific ecoregions based on a 29-year dataset. The horizontal axis represents months (e.g. 1 = January, 12 = December). The solid lines show the monthly-mean SST values. The dotted lines show the upper 95^th^ and lower 5^th^ quantiles of SST, which is a measure of within-month variation.

We used the following protocol to describe the nearshore SST patterns within an ecoregion. We pooled all 29 years of the derived SST values of mean monthly SST grid cells within an ecoregion and calculated the monthly-mean SSTs for each ecoregion, as well as a suite of other metrics derived from the monthly means as summarized in Figures 2 and 3. To evaluate spatial similarities in seasonal temperature patterns, we performed group hierarchical clustering on the ecoregions using these monthly SST metrics (n = 12 months×16 ecoregions). A permutation test (SIMPROF) was used to identify branches that were not significantly different (p<0.05). As a complement to the cluster analysis, the seasonal pattern of monthly-mean SSTs from each of the ecoregions was analyzed using nonparametric multi-dimensional scaling (nMDS). Euclidian distance was used to quantify the dissimilarity in seasonal temperature patterns among the ecoregions in the clustering and nMDS analyses. These analyses were conducted using the PRIMER software package v. 6.1.6 [25].

## Results

### Missing Data

**Figure 4:**
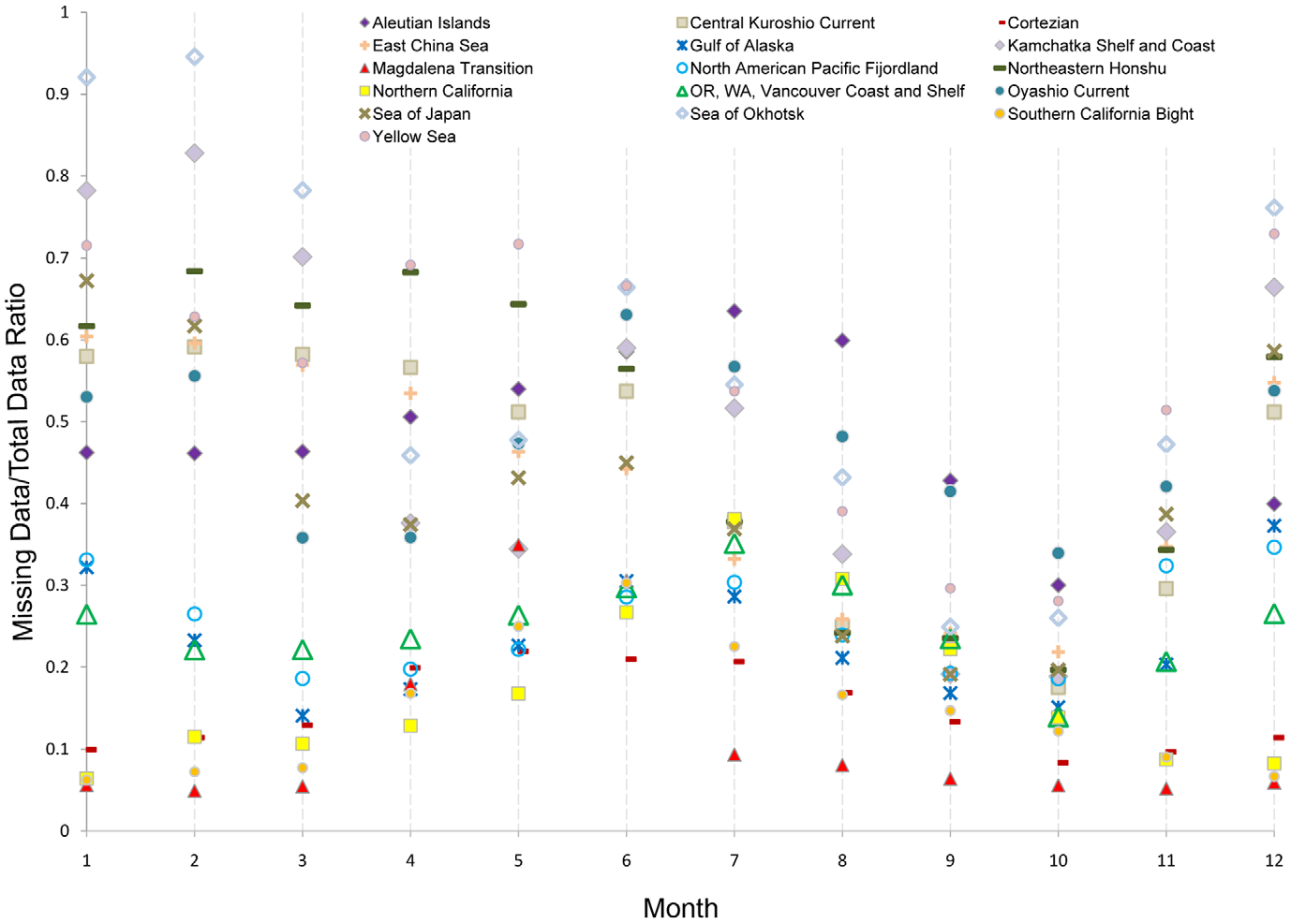
Fraction of missing data points for each month aggregated over the 29-year SST time series. Each individual month (e.g. all 29 Januarys) has over half-a-million possible SST point values. Although subarctic ecoregions tend to have a greater percent of missing data compared to lower-latitude ecoregions, at least 40,000 SST values meeting the quality criteria were present in the Temperate North Pacific study area for each of the months evaluated.

Several of the ecoregions had considerable missing data during some months. Ecoregions in the subarctic (e.g. Sea of Okhotsk), as well as other typically cloudy/foggy regions (e.g. Yellow Sea ecoregion and Oregon, Washington, Vancouver Coast and Shelf ecoregion, hereafter referred to as the “Oregonian”) can have upwards of 70% missing data for any one month (Figure 4). However, since the data included more than a half-million potential SST values per month, even 10% of the data yielded tens of thousands of values—enough to perform a robust analysis of ecoregional patterns. Although a concentration of acceptable SST values at one particular location within an ecoregion could potentially introduce a spatial bias, the data were examined visually, and it was determined that that was not the case.

### Long-term Mean SST and Annual Cycle

Figure 2 provides an illustration and description of the metrics used in our analysis, while Table S1 provides summary statistics for most of those metrics. The mean annual SST (29-year mean) among the Temperate North Pacific ecoregions varies from about 4°C in Okhotsk and Kamchatka ecoregions to a maximum of about 25°C in the Cortezian ecoregion (Table S1). There is a significant decreasing trend of mean annual SST with mean latitude (Pearson Product Moment Correlation, r = −0.94, p<0.001). The variance in SST is greater for Northwest Pacific ecoregions than in the Northeast Pacific (Table S1). The ecoregions with the greatest variance are the Yellow Sea and Sea of Japan, while those with the least variance are the Aleutian, Northern California, and Oregonian ecoregions. The mean annual cycles in SST in the North Pacific ecoregions are shown in Figure 3. The percent of variance in SST explained by the annual cycle varies from 31% for the Northern California ecoregion to 94% in the Yellow Sea ecoregion (Table S1). In the Northeast Pacific, the three ecoregions extending from Vancouver to Southern California had the lowest percent variance (ranging from 31 to 63%) explained by the annual cycle. For the remainder of the ecoregions in the Northeast Pacific, the amount of variance explained by the annual cycle ranged from 74% to 83%. In the Northwest Pacific, the East China Sea and Sea of Japan ecoregions had the lowest percent variance explained by the annual cycle (58% and 71%, respectively). For the remainder of the ecoregions in the Northwest Pacific, most (80–94%) of the variance in SST was associated with the annual cycle.

Within an ecoregion, the range of the annual cycle is defined by the difference between the minimum 29-year monthly-mean and the maximum 29-year monthly-mean SST (Table S1). Generally, the minimum monthly-mean SST in the Temperate North Pacific occurs in February and March, with the exception of the Magdalena and Northern California ecoregions which have minimum SSTs in April. The annual cycle temperature ranges are greater in the Northwest Pacific (mean range of 8 ecoregions = 15°C) as compared to the Northeast Pacific (mean range of 8 ecoregions = 7°C). The greatest annual cycle temperature range occurs in the Yellow Sea ecoregion, which has a range >23°C, while the Northern California ecoregion has the smallest range of about 3°C. In the Northeast Pacific, there is a smaller range in the annual cycle at mid-latitudes. A complementary analysis evaluated the number of months within each ecoregion falling within five temperature ranges (Table 1). For example, the Northern California ecoregion falls within only two of the five classes. By contrast, the Yellow Sea has months within all five classes.

**Table 1:** Number of months in various temperature intervals for ecoregions of the Temperate North Pacific.*

		Number of Months				
Province	Ecoregion	≤5°C	>5°C <12°C	≥12°C <20°C	≥20°C <25°C	≥25°C
**CTNEP**	Aleutian Islands	6	6	0	0	0
	Gulf of Alaska	4	7	1	0	0
	N. American Pac. Fijordland	0	9	3	0	0
	OR, WA, Vancouver	0	7	5	0	0
	Northern CA	0	1	11	0	0
**WTNEP**	S. CA Bight	0	0	10	2	0
	Cortezian	0	0	3	3	6
	Magdalena Transition	0	0	4	5	3
**CTNWP**	Sea of Okhotsk	7	4	1	0	0
	Kamchatka Coast	7	5	0	0	0
	Oyashio Current	6	4	2	0	0
	Northeastern Honshu	0	5	5	2	0
	Sea of Japan	0	6	4	2	0
	Yellow Sea	3	2	4	2	1
**WTNWP**	Central Kuroshio Current	0	0	5	4	3
	East China Sea	0	0	6	3	3

*The temperature intervals shown here, based upon monthly-mean SST values, have been previously identified as being critical for marine biota [45], [53].

The second type of temporal fluctuation experienced within each ecoregion is referred to as within-month SST variations, which is defined as the span of 5th and 95th quantiles and shown as the dotted envelopes in Figure 3. Of all the ecoregions the Sea of Japan ecoregion exhibits the highest within-month SST variation (12–15°C), across all months. In the Northwest Pacific, the Northeast Honshu and East China Sea ecoregions experience maximum within-month variation during the winter, with the East China Sea ecoregion exhibiting within-month SST variations of about 15°C. In contrast, the northern ecoregions in the Northwest Pacific (Kamchatka, Sea of Okhotsk, and Oyashio) have maximum within-month SST variation during the summer. The Central Kuroshio and Yellow Sea ecoregions have the lowest within-month SST variation in the Northwest Pacific. The magnitude of within-month SST variations is much less in the Northeast Pacific than in the Northwest Pacific. In addition, the seasonal patterns in within-month SST variations are less pronounced in the Northeast Pacific (with the exception of the Cortezian) as compared to the Northwest Pacific. In the Northeast Pacific, the Cortezian ecoregion exhibits large within-month variation (10°C) during the winter and minimal variation during the summer.

### Clustering Analysis

**Figure 5:**
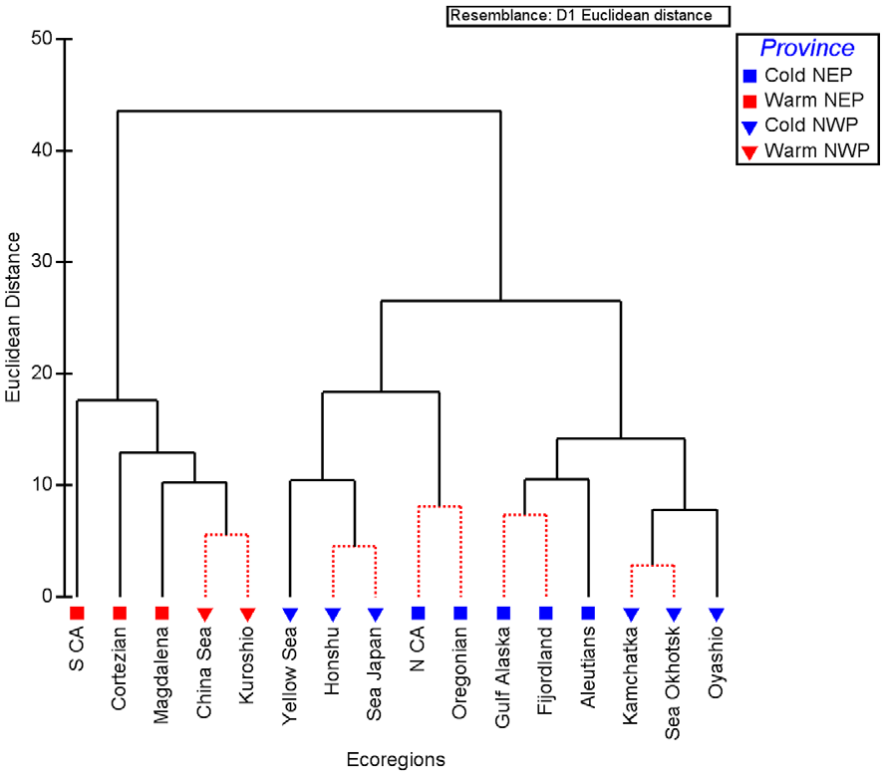
Hierarchical clustering dendrogram of 16 Temperate North Pacific ecoregions based on monthly-mean SST values. Clustering was performed with PRIMER [25] using 3000 iterations in SIMPROF. Distances are Euclidean. Red, dotted branches indicate no significant difference between linked ecoregions. NEP = Northeast Pacific; NWP = Northwest Pacific.

To evaluate similarity in temperature regimes, we performed an Euclidean-distance measure hierarchical clustering analysis based on ecoregion temperature means, medians, 5^th^ and 95^th^ quantiles, and ranges. In each case, the ecoregional SSTs group into two major branches (Figure 5) irrespective of what SST metric was used. These two major branches correspond to the Warm and Cold Temperate provinces on either side of the Pacific, splitting the MEOW ecoregions accordingly (Table S1). Within the warm temperate cluster, the Central Kuroshio Current and East China Sea ecoregions show no significant difference (p<0.05), while the Southern California ecoregion is the least similar of the five warm temperate ecoregions. Additionally, the Cortezian differs from Southern California and the rest of the warm temperate group. Finally, the Magdalena Transition is segregated from the Central Kuroshio Current-East China Sea grouping.

The cold temperate cluster splits roughly at 35°N into two secondary branches, which correspond to the northern and southern ecoregions within the Cold Temperate provinces. Within the southern sub-branch, the ecoregions partition to either side of the ocean basin. The Northern California and Oregonian ecoregions are not significantly different, nor are the Northeast Honshu and Sea of Japan ecoregions, while the Yellow Sea differs from both of these sub-branches. Within the northern sub-cluster, a further division separates the West and East Pacific locales, similar to the southern sub-cluster described above. To the east, the North American Pacific Fijordland and Gulf of Alaska ecoregions are not significantly different, though they do differ from the Aleutian Islands ecoregion. In the Western Pacific, the Kamchatka and the Sea of Okhotsk group together, separating from the Oyashio Current. An analysis using nMDS showed a very similar pattern to the clustering (not presented).

**Figure 6:**
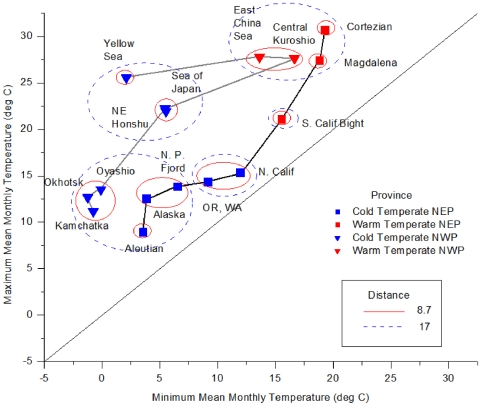
Ecoregions plotted by maximum monthly versus minimum monthly-mean SST over the 29-year record. The y = x line indicates where minimum and maximum temperatures within an ecoregion are identical. Hence, proximity to the line indicates minimal variability in seasonal monthly SST. The Euclidean distances of 8.7 and 17 were obtained through clustering analysis. Solid lines connect ecoregions in order of latitude along the NEP and NWP.

Figure 6 illustrates a different way to relate the thermal regimes by combining both the extent of similarity based on clustering of monthly-mean SST and temperature ranges (minimum and maximum monthly-mean SST). It is a unique way to visualize both geographic and temperature ranges simultaneously. Each ecoregion is plotted by the minimum and maximum monthly temperature observed over the 29-year record, with the solid line segments linking the ecoregions ordered by latitude on each side of the Pacific. The further offset an ecoregion is from the 45-degree line, the greater the range in the annual cycle. Highly variable ecoregions noted in Table S1 and Figure 3, such as the Cortezian and the Yellow Sea, deviate substantially from the line, while less variable ecoregions lay closer to the line. At the same time, similarity among ecoregions in overall seasonal patterns is captured by concentric ellipses representing different Euclidean distances that were derived from clustering on mean seasonal temperatures (Figure 5). In this case, Euclidean distances of 8.7 and 17 were chosen as they represent the maximum dissimilarity for any non-significant split (Gulf of Alaska and Pacific Fijordland) and the value that separates Southern California from the rest of the Warm Temperate ecoregions (Figure 5).

## Discussion

### Data uncertainties, challenges, and availability

Determining SST values in nearshore environments over a regional scale presented a significant challenge. Seasonal patterns in sea fog result in high levels of missing data, such as during the summer in the Yellow Sea [26] and Okhotsk ecoregions [27]. Upwelling regions, such as the Oregonian and Northern California ecoregions, also have missing data related to fog and cloud cover associated with strong thermal gradients between the land and coastal ocean [19], [28], [29]. Presence of sea ice also accounts for missing data, as the Sea of Okhotsk and Kamchatka Sea are partially covered with ice during winter months [30].

Moreover, failure of the AVHRR atmospheric correction to accurately deal with aerosol contamination due to Saharan dust storms and volcanic eruptions (e.g. Pinitubo in 1991) also led to long periods (months) of missing data [29], [31]. In addition, our decision to limit the SST data based on quality criteria and, to a much lesser extent, elimination of SST values <−2°C resulted in missing data. Due to varying data quality, there are differences in the extent of missing data between ecoregions and between months. For example, there is a high level of missing data in the Subarctic Northwest Pacific ecoregions during the winter (Figure 4). The worst case was Okhotsk in February with more than 90% of the data not meeting the quality standard. Conversely, 95% of the data values fell within acceptable quality limits in the Magdalena Transition ecoregion for the same month. In fact, mid-latitude ecoregions of the Northeast Pacific generally had good coverage year round (>80%). The abundance of data aggregated at the ecoregional scale overcomes the problem with missing data. Additionally, our SST analysis agrees with previous studies, such as large annual range in Yellow Sea [32] and small annual range in Northern California [33], suggesting that data are adequate for a seasonal and ecoregional analysis.

### Oceanographic Drivers

Sea surface temperature variations result from the interaction of heat exchange at the sea surface, circulation patterns, and mixing processes [33]. The primary circulation of the North Pacific is driven by the anticyclonic North Pacific Gyre (Figure 1), which consists of the swiftly-moving, poleward flowing, Kuroshio Current System (KCS) and the relatively slow-moving, equatorward flowing, California Current (CCS) connected by the East-West aligned North Pacific Current (NPC) and North Equatorial Current (NEC). The KCS and CCS are the main north-south boundary currents that largely regulate Pacific Ocean temperatures by transporting warm equatorial waters poleward by the KCS and vice versa by the CCS. The KCS is a deep, narrow, high-volume, swiftly-moving [34] western boundary current that extends from the East China Sea north to its confluence with the subarctic, south-flowing Oyashio Current (OC, [34]). Conversely, the CCS is a shallow (∼200 m), broad (∼1000 km), slow-moving [35] current that flows equatorward from the Gulf of Alaska, along the coasts of British Columbia to California [36]. At the point of Baja California, it turns westward, becoming the North Equatorial Current (NEC).

Nearshore SSTs within the CCS are also influenced by alongshore wind stress that result in high upwelling variability, especially in spring and summer [37], [38]. This upwelling results in the relatively constant year-round SSTs (Figure 3, Table S1) from Vancouver Island (Canada) to Southern California (USA). The warmer and more variable regions of SST in Baja California and the Cortezian result from sparse cloud cover at low latitudes and rapid solar heating [39]. In the west, the KCS features a more complicated ocean current system. Temperatures are governed by the confluence of the clockwise-flowing North Pacific Gyre and the anti-clockwise flowing Western Subarctic Gyre. The merging of warm, salty waters of the KCS from the south with relatively cooler, fresher waters of the OC and EKC from the north (Figure 1) generates a seasonally-complex SST pattern in the Northwest Pacific [34]. The OC-KCS convergence occurs off the east coast of Hokkaido, Japan, forming the Eastward-flowing Kuroshio Extension (KE, Figure 1), which infiltrates the inland seas of Japan and Okhotsk, creating a complicated and seasonally wide-ranging temperature pattern along their shores.

### Ecoregional Analysis

In this paper, we evaluated temperature patterns within coastal ecoregions in the North Pacific as defined in the MEOW biogeographic schema ([16], Figure 1), which offers several pragmatic advantages. First, aggregating the data by ecoregion mitigates the problems associated with missing data, particularly in the high latitude ecoregions. Second, several types of analyses were impossible or at least impractical with over 500,000 individual data points, such as clustering to reveal similarities and dissimilarities in temperature regimes along the coast. The third is that analysis at this scale reduces the effects of small-scale noise in the data. Ecoregional analysis also allows relating regional temperature patterns to large-scale distributional patterns of individual species and types of assemblages (e.g., coral reefs, kelp forests). While not the focus of this paper, overlaying species' biogeographic distributions on ecoregion temperature regimes allows the generation of a general thermal classification for a species (e.g., see [40]). Our decision to use the MEOW framework was based on the fact that MEOW is a biogeographic system for coastal marine areas based on taxonomic configurations and patterns of dispersal. Furthermore, MEOW allows for a more detailed ecoregional analysis than previous broader-scale schemes (e.g. [11], [41]), and is being utilized by ecologists evaluating regional patterns (e.g. [42]), the Ocean Biogeographic Information System (OBIS, http://iobis.org/home), and organizations (e.g., the “Non-indigenous Aquatic Species” Working group of the North Pacific Marine Science Organization). There are, of course, questions that cannot be addressed by summarizing SST by ecoregion, such as comparing the latitudinal rate of temperature change in the Northwest and Northeast Pacific or identifying the temperatures associated with the thermal breaks at the borders of an ecoregion (e.g., [5]). However, as mentioned, the processed data are provided [24] for such analyses.

The major aim of this effort was to evaluate regional temperature patterns in the North Pacific. In general, the mean annual SSTs (29-year mean) in this study are consistent with previous published studies from respective regions [33], [43]. One broad pattern found in this analysis is that the Northwest Pacific ecoregions at all latitudes experience a greater range in the annual cycle than do the Northeast Pacific ecoregions (Figure 3 and Table S1), which is consistent with previous studies [33], [43]. For example, the range of annual cycle was from about 3°C to 9°C in the Cold Temperate Northeast Pacific province ecoregions, compared to a range of 12°C to almost 24°C in the Cold Temperate Northwest Pacific province ecoregions. While less pronounced, a similar pattern was also observed in the Warm Temperate ecoregions, which show an annual range of about 6 to 12°C in the Northeast Pacific versus a range of about 11 to 14°C in the Northwest Pacific. The Yellow Sea experiences the largest range in annual cycle, which is attributed to the influence of the Asian monsoon [32]. Conversely, the nearshore waters of the Oregonian ecoregion show the smallest seasonal amplitude in the annual cycle range, similar to the findings of Wyrtki [33] and Yashayaev and Zveryaev [43].

Previous studies of SSTs in the central portion of the North Pacific basin have found that approximately 95% of the variance in SST is associated with the mean annual cycle and the percent variance associated with the mean annual cycle decreases near the coasts particularly in the Northeast Pacific [43]. In our analysis, the variance in SST associated with the annual cycle varied from 31% to 94%, and the annual cycle explained less of the variance in the northeastern ecoregions than for those in the northwestern ecoregions. It is not surprising that the amount of variance associated with the annual cycle were less than those in the central portion of the Pacific basin, since nearshore SSTs are influenced by coastal upwelling, riverine and land effects, and other nearshore processes.

Moreover, northwestern ecoregions generally experience greater within-month temperature variations than do northeastern ecoregions at approximately the same latitude (Figure 3). The within-month temperature variations are a result of both temporal and spatial variability in SSTs within an ecoregion. The steeper meridional temperature gradients within ecoregions in the Northwest Pacific as compared to the Northeast Pacific are likely responsible for a portion of this variation [33]. This is particularly likely in the Sea of Japan where within-month temperature variations can be as great as 15°C. In addition, the Japan/East Sea region is known for dramatic weather-system shifts that occur over the time-scale of a few days [44], which would increase within-month variation.

### Comparison of Ecoregional Temperature Patterns to Biogeographic Schema

Temperature is a driver of biotic distributions on regional and global scales [11], [45], [46], [47], thus we would expect some correspondence between the temperature patterns generated from the SST data and the patterns of the biologically-based MEOW provinces and ecoregions. Clustering on monthly-mean SSTs results in a primary division that corresponds unambiguously to the MEOW province categorizations of “Warm Temperate” versus “Cold Temperate” (Figure 5). Thus, biotic composition as inferred from the MEOW province boundaries may be related to nearshore SST. Furthermore, within each of the provinces, a number of ecoregions show distinct thermal regimes, supporting a biogeographical break. One example is the Southern California ecoregion that is markedly different from its neighboring Baja regions (Cortezian and Magdalena ecoregions). The dissimilarity is likely due to the circulation regime off the California coast; while upwelling is a critical factor in temperature mediation to the north, it is not as prevalent in the Magdalena and Cortezian ecoregions [35], [48]. Correspondingly, the range of mean temperatures in the Southern California Bight ecoregion is 3°C to almost 9°C smaller than the other ecoregions within the warm temperate cluster (Table S1). However, there was no significant difference in mean seasonal regimes with five pairs of neighboring ecoregions. One possibility is that some other component of the thermal regime other than the overall mean seasonal pattern drives the biogeographic patterns. For example, the occurrence of four months with a mean temperature <5°C in the Gulf of Alaska (Table 1) may be a key biological factor separating the biota in the Gulf from the North American Pacific Fijordland to the south. Alternatively, some factor(s) other than temperature may be important in generating distinct biotas between ecoregions, such as regional differences in primary productivity or effects of circulation patterns on larval dispersal. The last possibility is that the similarities in temperature patterns may indicate that the biotas in the neighboring ecoregions are not as distinct as suggested by the ecoregional demarcation. It is beyond the scope of this paper to evaluate these alternatives, but we suggest that further analyses of the biotic similarity in these ecoregions across a range of different taxa would be fruitful.

**Figure 7:**
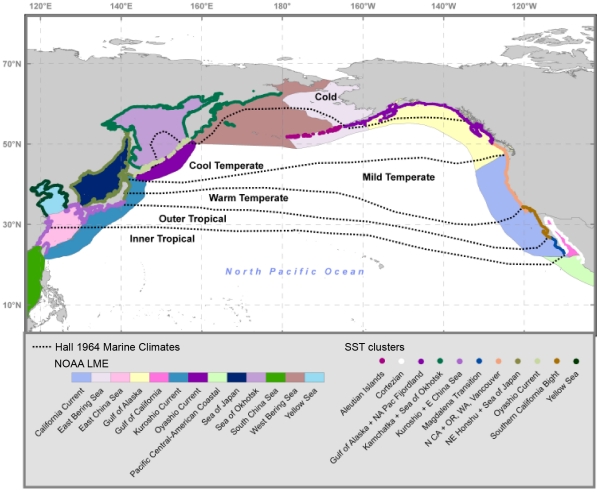
Boundary comparison among the thermal patterns based on our SST cluster analysis, marine climates of Hall [**40**], SST clusters based on cluster analysis, and NOAA's Large Marine Ecosystems of the World (LMEs). The LMEs are denoted by the outer colored areas. Hall's marine climates are indicated by the dotted black lines, while the SST clusters derived by clustering MEOW ecoregions by SST are delineated by the inner colored outlines.

It is also informative to compare SST patterns with the biogeographic schema defined by Hall [40] and by NOAA's Large Marine Ecosystems (LMEs) [41], [49]. The ecoregion clustering in Figure 7 is represented by combining MEOW ecoregions between which our clustering analysis found no significant differences (see Figure 5) into single entities that we term “SST clusters.” For example, the Oregonian and Northern California ecoregions group together to form a single SST cluster. It is clear that some of Hall's marine climate classifications in the Northeast Pacific match reasonably well with the clustering based on temperature. In the Northeast Pacific, Hall's “Outer Tropical” climate envelopes the Magdalena Transition; similarly, his “Warm Temperate” climate encompasses the Southern California Bight of the Warm Temperate Northeast Pacific province. Hall's “Mild Temperate” climate roughly corresponds to the SST cluster comprised of the combined Northern California and Oregonian ecoregions. However, Hall's “Cold” and “Cool Temperate” divisions do not agree as well with the subarctic SST clusters. Hall's “Cold” climate encompasses the Gulf of Alaska, Kamchatka, and Sea of Okhotsk ecoregions. Our results do not indicate that the Gulf of Alaska should fall within the “Cold” climate, as it has warmer minimum monthly temperatures than the Kamchatka and Sea of Okhotsk and its thermal regime does not differ from the North American Pacific Fjordland.

The correspondence of Hall's classifications in the Northwest Pacific is more complex. The Kamchatka and Sea of Okhotsk SST cluster falls completely within Hall's “Cold” marine climate, while the Oyashio ecoregion falls within Hall's “Cool Temperate” ecoregions, and has a thermal regime which differs from adjacent ecoregions. These breakouts appear generally consistent with our observed SST patterns. However, our cluster analysis found no difference between Hall's “Inner Tropical” and “Outer Tropical” climates in that Kuroshio and East China Sea ecoregions were not distinct in thermal patterns. Similarly, the Northeastern Honshu and Sea of Japan SST cluster that runs between the Pacific Ocean-facing sides of Honshu and Hokkaido Islands, spans Hall's “Warm Temperate” and “Mild Temperate” marine climate zones.

As shown in Figure 7, our SST clusters do not agree as well with LMEs, in particular in the Northeast Pacific. This difference is most apparent where the “California Current” LME combines the three distinct thermal SST clusters of the Northern California - Oregonian, the Southern California Bight, and the Magdalena Transition. As such, the “California Current” LME traverses the border between the Warm Temperate Northeast Pacific and Cold Temperate Northeast Pacific provinces. As seen in Table S1, there is a two-fold difference in SST range between southernmost (Magdalena) and northernmost (Oregonian) ecoregions contained within this single LME. Furthermore, significant differences between LMEs and the cluster-defined SST clusters of this analysis exist in the Northwest Pacific, where LME boundaries find similarities in different locations than indicated in our analysis. The SST cluster scheme lumps the East China Sea and Central Kuroshio Current ecoregions, which are separate according to the LME classification, while LMEs combine the Kuroshio and Honshu that we found had distinct thermal regimes. Some potential reasons for these discrepancies are that LME divisions are based on physiographic and trophic interactions [49] and encompass the entire shelf area compared to our nearshore analysis.

Our clustering efforts are based on nearshore SST within MEOW ecoregions, which are derived from a synthesis of previous biogeographic efforts [16]. In several cases, clustering of mean temperatures failed to pick up the differences on an ecoregional scale, that is, some neighboring ecoregions showed no significant difference when clustered based on mean SST. Comparison of the thermal patterns with the Hall and LME schemas also demonstrated several differences. As mentioned above there are several potential causes for differences between the temperature patterns and the biogeographic boundaries. These areas of major discrepancies deserve further study to evaluate the biotic reality of the boundaries and, assuming an ecologically realistic boundary, the cause(s) for biotic separation with neighboring regions with similar thermal regimes. In particular, all three biogeographic schemas have notable differences in the interface between the sub-arctic and arctic. This raises questions on how this interface should be defined, since this boundary is expected to shift poleward due to climate change [50].

### Potential Relationships to Climate Change

Nearshore SSTs will change in the future in response to climate change [46]. The North Pacific is especially vulnerable to environmental change, as it is reported to be warming 2–3 times faster than the South Pacific [51]. The present analysis and the processed data available in Payne et al. [24] provide nearshore temperature data against which to evaluate future measurements of SSTs in nearshore environments and a baseline on which to project potential climate change scenarios. Additionally, using the present regional temperature patterns, it is possible to speculate which ecoregions might be most susceptible to temperature increases, assuming that, in general, organisms living in areas with smaller temperature variations would be more susceptible to temperature increases (see [52]). Thus, the nearshore flora and fauna of Northeast Pacific ecoregions may be more susceptible to temperature increases than organisms in Northwest Pacific ecoregions, assuming an equivalent temperature increase. In particular, organisms in the Northern California ecoregion may be highly susceptible given the ecoregion's low annual temperature range, while organisms in the Aleutian ecoregion may be highly susceptible based on the ecoregion's low within month variation (Table S1, Figure 3). This speculation needs to be evaluated both by comparing the actual temperature ranges of organisms from field surveys and by evaluating temperature tolerances with experimental studies. Nonetheless, we suggest that analyses of existing temperature regimes can provide insights into what organisms and regions will be at the greatest risk from this aspect of climate change.

## Supporting Information

Table S1 **Summary statistics of sea surface temperatures (SSTs), including mean, minimum and maximum monthly mean, range of annual cycle, and variances in the Temperate North Pacific ecoregions.** The mean latitude and longitude for each ecoregion is shown in parentheses. CTNEP = Cold Temperate Northeast Pacific; WTNEP = Warm Temperate Northeast Pacific; CTNWP = Cold Temperate Northwest Pacific; WTNWP = Warm Temperate Northwest Pacific.
(DOCX)
