# Supplementary material for: Ecoregional Analysis of Nearshore Sea-Surface Temperature in the North Pacific
Source: PLoS One. 2012 Jan 11;7(1):e30105. doi: 10.1371/journal.pone.0030105 (PMC3256220; doi:10.1371/journal.pone.0030105)
Supplement: Table S1 — Summary statistics of sea surface temperatures (SSTs), including mean, minimum and maximum monthly mean, range of annual cycle, and variances in the Temperate North Pacific ecoregions. The mean latitude and longitude for each ecoregion is shown in parentheses. CTNEP = Cold Temperate Northeast Pacific; WTNEP = Warm Temperate Northeast Pacific; CTNWP = Cold Temperate Northwest Pacific; WTNWP = Warm Temperate Northwest Pacific. (DOCX) [file pone.0030105.s001.docx]

**Table S1. Summary statistics of sea surface temperatures (SSTs), including mean, minimum and maximum monthly mean, range of annual cycle, and variances in the Temperate North Pacific ecoregions.** *

|  | | | | **Temperature (°C)** | | | |  | |
| --- | --- | --- | --- | --- | --- | --- | --- | --- | --- |
|  | **Ecoregion** | **Latitude °N** | **Longitude °E** | **29-year Mean** | **Min. Monthly Mean** | **Max. Monthly Mean** | **Range of Annual Cycle** | **29-year variance (°C^2^)** | **% of variance explained by annual cycle** |
| **CTNEP** | Aleutian Islands | 47.7 - 55.2 *(52.6)* | 169.6 – -163.8 *(-172.3)* | 5.7 | 3.5 | 9 | 5.4 | 4.1 | 80.5 |
|  | Gulf of Alaska | 51.0 - 64.8 *(58.0)* | -164.7 – -136.9 *(-151.9)* | 7.4 | 3.8 | 12.6 | 8.7 | 11.5 | 83.2 |
|  | N. American Pac. Fijordland | 48.9 - 62.8 *(53.9)* | -139.1 – -120.9 *(-131.8)* | 9.5 | 6.5 | 13.9 | 7.4 | 8.2 | 82.6 |
|  | OR, WA, Vancouver | 40.4 - 50.3 *(45.9)* | -132.3 – -119.4 *(-124.9)* | 11.5 | 9.2 | 14.4 | 5.2 | 5.4 | 63.0 |
|  | Northern CA | 31.6 - 40.4 *(36.8)* | -127.7 – -116.9 *(-122.1)* | 13.5 | 12 | 15.4 | 3.4 | 4.2 | 31.1 |
| **WTNEP** | S. CA Bight | 24.2 - 36.9 *(30.8)* | -122.7 – -113.7 *(-116.6)* | 17.8 | 15.5 | 21.1 | 5.6 | 7.0 | 58.6 |
|  | Cortezian | 20.2 - 35.8 *(26.7)* | -115.8 – -102.0 *(-110.8)* | 24.8 | 19.3 | 30.7 | 11.5 | 23.2 | 77.8 |
|  | Magdalena Transition | 22.8 - 27.5 *(24.8)* | -115.7 – -109.8 *(-111.8)* | 22.6 | 18.8 | 27.4 | 8.6 | 12.3 | 74.1 |
| **CTNWP** | Sea of Okhotsk | 43.4 - 66.5  *(56.1)* | 131.6 – 168.4 *(148.9)* | 3.9 | -1.3 | 12.7 | 13.9 | 28.9 | 82.9 |
|  | Kamchatka Coast | 49.8 - 64.2 *(58.2)* | 157.1 – 179.9 *(166.6)* | 3.8 | -0.8 | 11.2 | 11.9 | 20.5 | 85.9 |
|  | Oyashio Current | 39.6 - 52.8 *(46.3)* | 142.7 – 160.6 *(149.9)* | 5.8 | -0.1 | 13.5 | 13.6 | 30.8 | 81.3 |
|  | Northeastern Honshu | 34.2 - 43.3 *(40.1)* | 139.6 – 145.4 *(141.4)* | 13.4 | 5.5 | 21.9 | 16.4 | 36.2 | 79.8 |
|  | Sea of Japan | 34.3 - 53.2 *(42.4)* | 125.6 – 142.9 *(136.8)* | 12.8 | 5.5 | 22.3 | 16.8 | 59.1 | 58.2 |
|  | Yellow Sea | 32.2 - 43.6 *(37.5)* | 113.1 – 128.0 *(122.2)* | 13.6 | 2.1 | 25.6 | 23.5 | 71.2 | 93.5 |
| **WTNWP** | Central Kuroshio Current | 28.3 - 36.2 *(33.4)* | 128.5 – 143.7 *(134.0)* | 21.5 | 16.7 | 27.7 | 11.0 | 17.7 | 80.6 |
|  | East China Sea | 22.7 - 35.9 *(30.3)* | 113.5 – 130.7 *(124.3)* | 20.3 | 13.6 | 27.8 | 14.2 | 31.8 | 70.7 |
|  |  |  |  |  |  |  |  |  |  |

*The mean latitude and longitude for each ecoregion is shown in parentheses. CTNEP = Cold Temperate Northeast Pacific; WTNEP = Warm Temperate Northeast Pacific; CTNWP = Cold Temperate Northwest Pacific; WTNWP = Warm Temperate Northwest Pacific.
